# Understanding a Care Management System’s Role in Influencing a Transitional-Aged Youth Program’s Practice: Mixed Methods Study

**DOI:** 10.2196/39646

**Published:** 2022-12-16

**Authors:** Rubina F Rizvi, Courtney B VanHouten, Van C Willis, Bedda L Rosario, Brett R South, Megan Sands-Lincoln, David Brotman, Jeffery Lenert, Jane L Snowdon, Gretchen P Jackson

**Affiliations:** 1 IBM Watson Health Cambridge, MA United States; 2 Department of Pediatric Surgery, Vanderbilt University Medical Center Nashville, TN United States; 3 Department of Pediatrics, Vanderbilt University Medical Center Nashville, TN United States; 4 Department of Biomedical Informatics, Vanderbilt University Medical Center Nashville, TN United States; 5 Intuitive Surgical Sunnyvale, CA United States

**Keywords:** care management solution, foster care youth, mixed methods study, interviews, qualitative data, quantitative data, process improvement

## Abstract

**Background:**

Extended foster care programs help prepare transitional-aged youth (TAY) to step into adulthood and live independent lives. Aspiranet, one of California’s largest social service organizations, used a social care management solution (SCMS) to meet TAY’s needs.

**Objective:**

We aimed to investigate the impact of an SCMS, IBM Watson Care Manager (WCM), in transforming foster program service delivery and improving TAY outcomes.

**Methods:**

We used a mixed methods study design by collecting primary data from stakeholders through semistructured interviews in 2021 and by pulling secondary data from annual reports, system use logs, and data repositories from 2014 to 2021. Thematic analysis based on grounded theory was used to analyze qualitative data using NVivo software. Descriptive analysis of aggregated outcome metrics in the quantitative data was performed and compared across 2 periods: pre-SCMS implementation (before October 31, 2016) and post-SCMS implementation (November 1, 2016, and March 31, 2021).

**Results:**

In total, 6 Aspiranet employees (4 leaders and 2 life coaches) were interviewed, with a median time of 56 (IQR 53-67) minutes. The majority (5/6, 83%) were female, over 30 years of age (median 37, IQR 32-39) with a median of 6 (IQR 5-10) years of experience at Aspiranet and overall field experience of 10 (IQR 7-14) years. Most (4/6, 67%) participants rated their technological skills as expert. Thematic analysis of participants’ interview transcripts yielded 24 subthemes that were grouped into 6 superordinate themes: study context, the impact of the new tool, key strengths, commonly used features, expectations with WCM, and limitations and recommendations. The tool met users’ initial expectations of streamlining tasks and adopting essential functionalities. Median satisfaction scores around pre- and post-WCM workflow processes remained constant between 2 life coaches (3.25, IQR 2.5-4); however, among leaders, post-WCM scores (median 4, IQR 4-5) were higher than pre-WCM scores (median 3, IQR 3-3). Across the 2 study phases, Aspiranet served 1641 TAY having consistent population demographics (median age of 18, IQR 18-19 years; female: 903/1641, 55.03%; race and ethnicity: Hispanic or Latino: 621/1641, 37.84%; Black: 470/1641, 28.64%; White: 397/1641, 24.19%; Other: 153/1641, 9.32%). Between the pre- and post-WCM period, there was an increase in full-time school enrollment (359/531, 67.6% to 833/1110, 75.04%) and a reduction in part-time school enrollment (61/531, 11.5% to 91/1110, 8.2%). The median number of days spent in the foster care program remained the same (247, IQR 125-468 years); however, the number of incidents reported monthly per hundred youth showed a steady decline, even with an exponentially increasing number of enrolled youth and incidents.

**Conclusions:**

The SCMS for coordinating care and delivering tailored services to TAY streamlined Aspiranet’s workflows and processes and positively impacted youth outcomes. Further enhancements are needed to better align with user and youth needs.

## Introduction

### Foster Care

Extended foster care is considered an effective intervention to help prepare young adults to transition into adulthood to live independent, healthy, and successful lives and to help mitigate the risk of undesirable outcomes. However, providing extended foster care is a complex process and requires coordination and collaboration among various social service departments and stakeholders. The c*omplex social reality* of problems encountered in social work is seen universally [1]. Often, youths in foster care face multiple issues at a single point in time with competing priorities around when and how to address them [2]. A robust and integrated system of care is needed to understand the optimal processes and services to provide support for each individual youth with unique needs [3]. According to child welfare policy and services experts, the complex task of managing youth in foster care has been considered similar to the role of a parent and hence called “corporate parenting” [4].

The transition to adulthood is a critical stage in one’s life. A young adult may encounter multiple significant challenges in accessing the required resources and developing the skills essential to becoming self-sufficient. Transitional-aged youths (TAYs) are young people between the ages of 16 and 24 years [5] who are in transition from state custody or foster care environments and are at risk. Such individuals are at high risk of experiencing several undesirable outcomes, such as mental health or behavioral disorders [6], economic insecurity [7], housing instabilities [8,9], criminal justice involvement [8], and unintended pregnancies [10]. Aspiranet, one of California’s largest social service organizations with headquarters in San Francisco, California, provides a bridge between foster care and independence by providing extended foster care services to enable TAY with the necessary life skills, counseling, and medical treatment. Extended foster care can help protect TAY from some of these adverse outcomes [11].

### Foster Care Challenges

Foster program–specific challenges, such as large program sizes, high employee and benefit recipient turnover, disjointed care coordination, and complex health and emotional needs in foster children, make this already challenging task especially difficult. In the United States ≥400,000 children and youths are in the foster care system, with more than 150,000 youths aged 14 to 21 years enrolled in 2017 [12]. According to the Adoption and Foster Care Analysis and Reporting System data, approximately 20,000 youths age out of foster programs each year [13]. In California, the foster care population is the largest in any state, with approximately 51,000 children in foster care and a foster care rate of 5.8 per 1000 children using the 2019 US Census Bureau data, which further compounds the challenge [14]. TAYs in foster care programs need tailored attention and action plans to address their individual and often multiple needs according to the California Youth Transitions to Adulthood Study [15]. Overall, almost one-third (30.9%) of the youth reported being homeless sometime during the 5-year study period [16]. Almost 53% (n=404) of California Youth Transitions to Adulthood Study participants were found to have a positive diagnosis for one or more current mental and behavioral health disorder, with a greater frequency of diagnosis among females than males (57.5% and 46.9%, respectively) [17]. Overall, about 44% of youths had justice system involvement before the age 17 interview [18].

The complexity of social care services among extended foster care youth, along with siloed services and a lack of integrated information systems, leads to less-effective and less-efficient care delivery and communication [19]. To address this gap, the establishment of data governance and management, streamlining of processes, and integration of disparate systems under a common platform are needed [20,21].

### Care Management Solutions

Care management programs and systems have been widely adopted in health care provider organizations, and emerging evidence suggests that technologies to support care management may provide value in delivering complex care across social services outside the clinical environment. As reported in earlier studies, an advanced technology-based tool could enhance the care delivery process through the integration of data- and information-coordinated care. The benefit of such a tool ranges from providing comprehensive services to various stakeholders across governments, nonprofits, and other organizations to addressing the challenges of information silos and limited resources [19,22].

Although evidence supports the value of case management systems in stabilizing vulnerable adult citizens, to our knowledge, such technologies have not yet been examined in the TAY population. We sought to investigate whether and how a cloud-based social care management solution could improve the care of transitional youth in a large foster care program using a mixed methods approach. We hypothesized that the care management system would not only streamline workflows and facilitate social care service providers in delivering more effective and efficient care but also improve youth outcomes and successful transition into a self-sufficient adulthood.

## Methods

### Study Site

Aspiranet is one of California’s largest nonprofit social service organizations providing support for children, youth, and families [23,24]. Founded in 1975 with a single group home, Aspiranet now runs 44 community-based sites across the state. It provides a spectrum of services such as foster care and adoption support, residential group home care, support for youth making the transition from foster care to adulthood, mental and behavioral health services, intensive home-based care, and community-based family resources [25]. More than 35,000 children, youths, and parents have benefited from Aspiranet’s services.

### Care Management System

In 2016, Aspiranet adopted IBM Watson Care Manager (WCM) to transform its social services programs from a traditional, paper-based process to an integrated and interdisciplinary technology-based system. WCM and IBM Health and Human Services Connect360 (data hub) is an integrated and configurable cloud-based software-as-a-service solution that allows teams to collaborate across agencies and reduce silos and provides a holistic view of the person from a historical and program perspective [26].

WCM enables child welfare workers to develop integrated care plans, triage vulnerable residents to the services, and foster collaboration with other care professionals (eg, within and external to Aspiranet, such as outpatient and school-based mental health services) to optimize service delivery. WCM integrates the Casey Life Skills (CLS) tool kit to help guide and assess the independent skills that youths need to achieve their long-term goals [27]. CLS assesses life skill domains built on the principles of psychometric measurement that defines a life skills assessment not as behavioral performance but as a general life skills ability. Aspiranet fully implemented WCM over the course of 2 years (2016-2018).

### Study Design and End Points

#### Overview

A mixed methods study was conducted to understand the impact of WCM on Aspiranet’s workflow processes and youth outcomes. The primary study end points were both qualitative and quantitative measures. Qualitative examples included Aspiranet employees’ perspectives on the impact of new technology on care management effectiveness and efficiencies compared with previous practices. We also looked at quantitative measures including program outcomes such as a change in school enrollment, employment and housing status, number of undesired events, and user satisfaction scores (5-point Likert scale) pre- and postimplementation. Qualitative data were obtained from interviews with Aspiranet employees who were WCM users. Quantitative data were extracted from reports, backend use logs, and data repositories. The study was conducted from January 1, 2014, to March 31, 2021, with data collection divided into 2 main phases: before using WCM (before October 31, 2016) and after using WCM (November 1, 2016, to March 31, 2021).

#### Study Participants

Purposive sampling in collaboration with Aspiranet administrative staff was used to recruit Aspiranet employees (ie, leaders and life coaches) to participate in the study. Aspiranet leaders were executives in various roles, and life coaches were the case managers who provided supportive services to the at-risk youth transitioning to adulthood at various Aspiranet locations. All participants were aged ≥18 years, who had experience in administering the TAY program both before and after the implementation of WCM. Informed consent was obtained from each participant.

### Ethics Approval

All study participants provided written consent to participate in personal audio-recorded interviews. This study was deemed exempt from human subjects research regulations by the Western Institutional Review Board (WCG IRB Work Order #1-1437184-1), exempted under 45 CFR § 46.104(d). All key study personnel involved in the design or conduct of the research completed the required education and certification on the protection of human research subjects.

### Qualitative Data: Semistructured Interviews

In-depth interviews were conducted over videoconferencing on WebEx using a semistructured topic guide (Multimedia Appendix 1) to elicit information about fostering collaboration, optimizing care management for TAY, workflows, the experience with WCM and legacy systems, benefits and challenges of WCM, and the impact on efficiency and youth-centered care coordination. Interviews were conducted by a physician informatician (RFR) and medical ethnographer (CBV) with training and expertise in evaluating user experience and the effects of health information technologies.

### Quantitative Data: Program Outcomes

#### Overview

Pre-WCM secondary data were primarily collected from the Aspiranet Legacy Data Hub containing higher-lever descriptive data about employees and enrolled youth. Owing to inherent barriers associated with management and tracking, no paper-based data were collected. Post-WCM data were collected from both the Aspiranet Legacy Data Hub and a Cognos reporting system (post-WCM implementation), describing a more granular picture of WCM use and program outcomes; for example, goals, actions, and tasks. For the data coming from legacy databases, the technical staff at Aspiranet performed data extraction, cleansing, and quality checks, which were validated by 2 IBM researchers (BRS and RFR). For the Cognos data, an IBM IT specialist worked closely with the Aspiranet staff to run the reports. Deidentified backend data for 2 specific periods, pre- and post-WCM implementation, were obtained for analysis.

The data collected included the number of enrolled TAY, the number of goals established (ie, a set achievement to reach a milestone, eg, getting a driver’s license or enrolling in college), tasks (ie, actions taken to reach that specific goal, eg, pass a permit test or file a college application), and the number of incidents or undesired events (eg, car accident, school suspension, and police involvement).

#### Qualitative Analysis

Interview transcripts were transcribed, deidentified, and analyzed by 2 members of the research team (RFR and CBV) using NVivo software (QSR International) [28,29]. A thematic approach directed by grounded theory [29] was used to develop a codebook in which disagreements were resolved through discussion until consensus was reached.

Coding in qualitative analysis is a process of systematically categorizing qualitative data into themes and patterns. The process entails open coding (ie, first breaking down textual data into discrete parts), followed by axial coding (ie, drawing connections between codes), and finally selective coding (ie, cataloging all categories together around 1 core theme) [30]. Coding was performed sequentially by 2 research team members (RFR and CBV), with regular communication and discussion to ensure consistency and accuracy. The perspectives of leaders and life coaches were collected about pre- and post-WCM experiences, such as the impact on workflows, quality of service delivery, and youth outcomes.

#### Quantitative Analysis

Descriptive statistics for each outcome of interest were summarized as frequencies (percentages) for categorical data or as mean (SD) or median (IQR) for normally distributed or nonnormally distributed continuous data, as appropriate for pre- and post-WCM periods. Examination of normal distribution assumptions for continuous data was determined using quantile-quantile plots and histograms. These plots are graphical ways to check whether the data follow a normal distribution. All statistical analyses were conducted using SAS language on WPS Analytics (version 4.2; World Programming) [31].

## Results

### Overview

The mixed methods study results are provided in subsequent sections. Qualitative analysis of the primary data is presented first followed by a quantitative analysis of the secondary data.

The results validated how the management of vulnerable populations, such as TAY, is multifaceted and has been operating across various fragmented, siloed systems until the implementation of WCM in 2016. Qualitative analysis of primary data is presented first, followed by quantitative analysis of secondary data, highlighting how WCM is facilitating the streamlining of care processes and the resulting outcomes.

### Primary Data: Qualitative Analysis

A total of 6 Aspiranet employees agreed to participate in the study. Table 1 summarizes the participants’ characteristics. All were aged >30 years (median 37, IQR 32-39) and have either a bachelor’s or master’s degree, and the majority (5/6, 83%) were females. Participants included leaders (4/6, 67%) and life coaches (2/6, 33%) having a median of 6 (IQR 5-10) years of experience at Aspiranet and overall experience in this field of 10 (IQR 7-14) years. Regarding self-assessed technology skills, most (4/6, 67%) participants rated themselves as experts or as close to an expert. The median time spent on each interview was approximately 56 (IQR 53-75) minutes.

**Figure 1:**
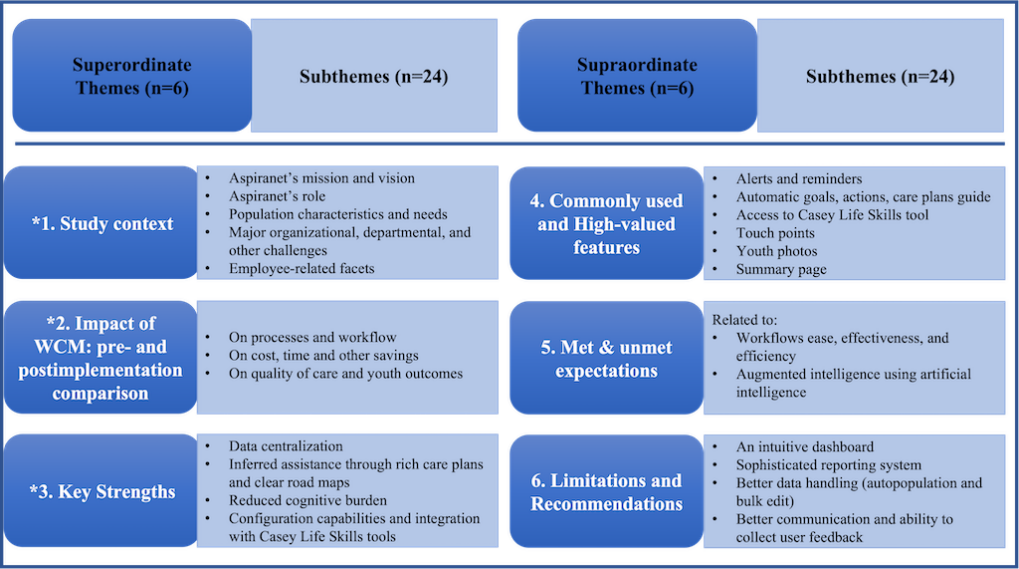
Thematic analysis reporting superordinate and subthemes (*key themes). WCM: Watson Care Manager.

The thematic analysis of 6 transcripts by 2 qualitative experts (RFR and CBV) unveiled various subthemes (n=24) that were grouped into the following higher level superordinate themes (n=6), as summarized in Figure 1.

The 6 superordinate themes and the corresponding subthemes that emerged from the thematic analysis of the interview transcripts are described in subsequent sections.

**Table 1:** Study participant characteristics (n=6).

ID	Gender	Age (years)	Degree	Role	Experience at Aspiranet (years)	Overall Experience (years)	Self-assessed technology skill level	Interview duration (minutes)
1	Female	37	Master’s	Life coach	5	7	Intermediate	52:52
2	Female	32	Bachelor’s	Life coach	6	10	Expert	67:02
3	Female	31	Master’s	Leader	3	5	Expert	48:03
4	Female	63	Bachelor’s	Leader	10	10	Close to an expert	54:43
5	Male	39	Bachelor’s	Leader	12	15	Between intermediate and expert	75:37
6	Female	36	Master’s	Leader	6	14	Close to an expert	58:28

### Superordinate Theme: Study Context

This theme has subthemes that are explained in subsequent sections.

#### Aspiranet’s Mission and Vision

The mission of Aspiranet’s TAY program is to provide young adults between the ages of 18 and 24 years with a level of support and services so they can be successful once they transition out of foster care and start living on their own. The TAY program’s long-term vision is to help support a world in which all children, youths, and their families are loved and cared for and that they have the resources that they need to lead a healthy and successful life.

This is a complex challenge. Aspiranet’s life coaches help link their clients with governmental social resources, while also helping them develop the skills needed to achieve basic life skills (eg, housekeeping) and supporting their path to achieving and maintaining safety, permanency, and economic success (Textbox 1). In addition to the diversity of client demographics (eg, age, race, ethnicity, and religion), life coaches may help address a range of issues from emotional, behavioral, and mental health conditions to teen pregnancy, homelessness, and employment challenges. Life coaches’ clients often require dramatically different levels and types of assistance.

**Aspiranet staff feedback on their mission and vision.**

- Aspiranet’s mission
  “I think, (the role of) Aspiranet is to provide our clients with the foundation & support to be able to kind of live on their own & provide resources, (that could guide) them to transition from foster care to a more independent living.” [Life coach]
- Aspiranet’s vision
  “But, I mean, our vision is a world in which all of our children, & for my division is youth are loved & cared for, you know, & that they have the resources that they need to try.” [Leader]

#### Aspiranet’s Role

The role of Aspiranet starts with first breaking down communication barriers with the youth who often come in with multiple traumatic experiences, rendering them emotionally unstable. This leads to an open and more productive discussion between the youth and caregivers and helps to identify the youth’s specific needs (education, housing, employment, and basic life skills) and to make targeted plans. Aspiranet plays an important role to help youth achieve the actual goal by linking resources to needs through collaboration with various agencies (Textbox 2). To make changes at the macro level, Aspiranet plays an essential role in engaging with the legislative process, particularly at the state level:

**Aspiranet staff feedback on their role with at risk youth.**

- “I was going to say, it’s just kind of my job is kind of like the tour guide. It’s like, I’m like that step. Yeah, like on their side so, like, as they’re kind of moving along, I’m literally there through every step.” [Life coach]

#### Population Characteristics and Needs

All interviewees mentioned the prevailing diversity in youth population demographics in terms of gender, age (18-24 years), race and ethnicity, and religious backgrounds. All patients experienced some type of trauma in their lives. They all pointed out an array of pressing challenges that their clients face (Textbox 3), often more than one at a time. Examples include age-related emotional challenges, behavioral and mental health conditions, housing, education, employment challenges, and lack of basic life skills as they are often systemized (ie, coming from structured facilities with more dependency on others), multiple placements and acquaintance to environment and individuals, and pregnancy at a young age:

**Aspiranet staff feedback on challenges that their incoming youth encountered.**

- “I would say housing just because the market for this particular population is scrutinized sometimes only because they’re young, so when it comes down to being 21, a lot of apartment complexes don’t necessarily like to work with 21 year old. Landlords think our clients are not mature enough to handle the pressure of being on their own. So, it’s a lot of the housing & on top of them having the housing crisis that we’ve had for a few years.” [Leader]

#### Major Organizational, Departmental, and Data Location or Complexity Challenges

Several challenges were brought up by leaders and life coaches and grouped into 2 main categories: one independent of the COVID-19 pandemic and the other ensuing or surfacing during the COVID-19 pandemic. Agencies working in silos owing to lack of integration and coordination are another important challenge. The continuing reliance on paper and not being fully digitalized added more challenges to the processes. Some challenges noted include having large amounts of data from different data resources; data existing in various formats and in different locations; duplicate data entry; and not being able to comprehend the full picture of the youth’s records from multiple discrete pieces of data. Financial challenges and limited funding, especially to meet housing needs in the state of California with the rising cost of living, were mentioned most often. Among other challenges that are directly or indirectly affected by the COVID-19 pandemic are those related to hiring staff, developing and training new employees, and excessive workloads. (Textbox 4)

**Aspiranet staff feedback on their day-to-day challenges.**

- Lack of integration and coordination
  “You know, the lack of integration or coordination. With other departments & agencies, we often work in silos where other providers are out there, providing the same type of work that we’re providing & kind of doing it in silos.” [Leader]
- Reliance on paper-based data
  “...another issue is just in terms of the integration of services, it’s really this way of working where we’re still very reliant on paper...” [Leader]
- Fragmented data (in discrete formats and locations)
  “...(we) collect a lot of data associated with both our youth as well as the services we’re providing. Then (there is data) on an ongoing basis (such as) case notes, service plans, all sorts of things... for each youth that we serve &...lot of cases, those pieces of information are in lots of different locations or different systems.” [Leader]
- Challenges associated with the pandemic
  “Staff development, the workload being too high, insufficient time, specifically over the last year during the pandemic.” [Life coach]

#### Employee-Related Facets

An employee’s overall integrity and commitment to help clients achieve their goals is considered an indirect measure of their performance. Therefore, it is the quality and not the quantity of work that steers the performance evaluation (Textbox 5). All employees mentioned that the ongoing training they periodically receive helped them better understand the processes and better use available tools, particularly WCM:

**Aspiranet staff reflect on their performance related measures.**

- “At least from what I’ve been able to see, and how I evaluate the employee, it is not really about the quantitative work, (it is) more about the richness of the work.” [Leader]

### Superordinate Theme: User-Reported Benefits of WCM

This theme has subthemes that are explained in subsequent sections.

#### Impact on Workflow Processes

Beyond the convenience of access and minimizing the gathering of paperwork, all respondents brought up the benefits of having a centralized electronic system in which data were entered and saved in real time. Rather than entering weekly contact notes (before the WCM), notes are being entered in a timely manner without waiting for weekly data dumps. Data could now be retrieved and viewed immediately from anywhere, anytime, and anyone from the team.

WCM enhanced the usability and utility of youth information through the ability of configuration capabilities to retrieve and present desired reports. The participants discussed the importance of effective and efficient workflows and how this efficiency enabled them to rapidly triage vulnerable residents to the services they need in a quicker manner. This ease of access to a full synopsis of any client allows for flexibility in the performance of client duties. In an emergency, a life coach unfamiliar with a client could simply look up all their pertinent information. Although not a perfect solution, this capability did provide a significant improvement over the current workflow (Textbox 6).

**Aspiranet staff reflect on the transition before and after implementation of Watson Care Manager.**

- Real leader time electronic data entry
  “Prior to WCM, (the life coaches) had to write down notes then go to the file cabinet to pull out notes or to put them into a file & so now with Watson, the notes get entered in real time... as they’re having their interaction with the young adult, they’re already typing in the notes.” [Leader]
- Effective or timely care delivery
  “...(the process is) transitioning...to a real time information mindset. In other words, the data isn’t updated once a week, or once a quarter. It is updated once a day; it's updated as frequently as it needs to be...Personally, I think that's a huge shift in the way people work & I think it’s a huge shift in the way people are able to kind of better manage their youth relationship.”
- Easy access to data
  “Before, you know, we just had it (paper records) in a chart room. You had to be literally in the office to find anything you wanted. So, it’s really great to be able to have that on hand, made it smoother & when, in emergencies or in crisis where you need to access that information quickly.” [Life coach]

#### Impact on Time and Cost Savings

Participants believed that there were definite time savings realized after WCM implementation that indirectly or directly resulted in cost savings. Some of the reasons included saving time on commute due to convenient access to the tool, less overtime granted to catch up on notes, and more effective notes retrieval (Textbox 7).

**Aspiranet staff identify how Watson Care Manager potentially affected time savings.**

- Time savings
  “Case so absolutely, I would say a savings in the travel & the time in printing & scanning & in having to set up meetings because now it’s more collaborative & people are more up to speed or the cases.” [Leader]
- Cost saving as proxy of time savings
  “Because a lot of overtime was granted catching up on notes... there’s been tons of time savings... Watson is available on electronic devices. It’s mobile. They don’t have to come back to an office to write down their notes or to type things, & they’re doing it out in the field. I’m 100% positive that it has saved our life coaches a lot of time.” [Leader]

#### Impact on Quality of Care and Outcomes

According to the participants, their interactions with youth are much more enriched; that is, youths are more focused and engaged while managing their own care plans. There is consensus that youth outcomes are also improving, such as better retention, which could be further validated by examining objective data. However, the reason for these improvements may be multifactorial because WCM is only one of several modifications to workflows (Textbox 8).

**Aspiranet staff discuss how Watson Care Manager impacted their work with the youth.**

- Enriched interactions and better engagement
  “Definitely, I could see (improvement), like, it kind of helps to enhance in a sense because, like I said, the youth can more physically see what’s going on...so that does help because it’s (they) more focused...” [Life coach]
  “...but I think definitely (there is an improvement)...but from my observation (there is) an increase in youth engagement.” [Leader]
- Better outcomes
  “...[There is]decrease in voluntary & involuntary discharges. Some of the data that I’m seeing [outcomes] right now can I tie it to Watson 100% confidently? No! but I think that there’s a correlation.” [Leader]

### Superordinate Theme: Key Strengths

This theme has 4 subthemes explained in subsequent sections with supporting quotes.

#### Data Centralization

The tool served as a convenient, centralized system, with all youth information consolidated in one place, thus providing a holistic 360° view of any youth at any time (Textbox 9).

**Aspiranet staff discuss how Watson Care Manager consolidated their workflow.**

- Centralized data hub
  “Oh, yeah, having Watson Care Manager keeps everything in the centralized system. That way we don’t have to go back and forth in between pulling up the youth’s goals in their folder. We have it all in our Watson Care Manager.” [Leader]
- Holistic view of youth profile
  “...here again, this kind of a 360 degree view that’s accessible to any member of a care team at any point.” [Leader]

#### Inferred Assistance Through Rich Care Plans and Clear Road Maps

WCM helped all youth receive high-quality care, regardless of the complexity of the youth’s needs or the experience level of the life coach by rendering rich care plans with clear road maps. As stated earlier, many life coaches had heavy client burdens, and leaders found it difficult to hire new staff in the wake of the COVID-19 pandemic. As such, life coaches had large client loads, and a number of coaches had few years of experience. The large client list and complexity of each client case meant that each client required an array of assessments, documents, and other appraisals to ensure their health and safety (Textbox 10). By embedding access to decision support, WCM supported life coaches with varying levels of experience to perform treatment protocols developed by seasoned veterans:

**Aspiranet staff reflect on how Watson Care Manager assisted them in complex tasks.**

- “Say, if I’m putting in a safety plan, it will actually alert me to ask ‘did I contact any medical needs? Are the police involved?’ It (WCM)reminds me ‘hey, you need to check on these things’... So, you’re not like trying to go off of experience... I think really guides our best practice.” [Life coach]

#### Reduced Cognitive Burden

In this capacity, WCM functioned as a “memory bank,” ensuring that no tasks or actions needing attention are overlooked or missed action (Textbox 11). This is provisioned through the WCM capability of alerts and prompts, in addition to real-time data entry, eliminating any recall biases while entering data:

**Aspiranet staff reflect that Watson Care Manager would aid them in the full completion of tasks.**

- “Watson will project for specific actions that need to be done to that task. So, say, for instance, if I put in a safety plan, it’s going to prompt me (and ask) have you done this, this, this and this? So instead of me trying to operate on memory, Watson’s actually projecting and saying, ‘stop! -do this right now or else’.” [Life coach]

#### Configuration Capabilities and Integration With CLS

The integration of WCM with the CLS tool has provided us with an opportunity to follow youth progress in a more intuitive and efficient manner. Earlier, the role of the CLS tool was more for compliance checks but now it is being used more meaningfully (Textbox 12):

**Aspiranet staff feedback on the intuitive integration of Casey Life Skills into their workflow.**

- “I am very excited about the configuration within Watson to our Casey life skills tool. Traditionally, we use this tool and (it was) just filed away... it was more transactional compliance check, completed it every 6 months, and it was thrown into the file. (But) With the configuration that we did within Watson recently, we were able (have) the scores added to the thing in Watson and then we’re able to pull a report from Cognos to be able to demonstrate growth.” [Leader]

### Superordinate Theme: Commonly Used or High-Valued Features

The users mentioned several commonly used features. A few that were brought up repeatedly are alerts and reminders, automatic goals or action, configurability with CLS, touchpoint notes, youth photos, and the summary page (Textbox 13).

**Aspiranet staff feedback on their most commonly used Watson Care Manager features in their workflows.**

- Alerts and reminders
  “Watson so that we get alerted when the system recognizes. Someone may benefit for mental health services so that the life coach, and the team is aware, and can provide those referrals to the community to address any gaps.” [Leader]
- Automatic goals, actions, and care plans
  “So, definitely the automatic goals action alerts. I think really guide our best practice and are used the most often.” [Leader]
- Configurability with Casey Life Skills
  “Casey’s (CLS) are actually interacting with the goals so that’s really nice. That’s a new feature that’s just kind of been developing. And so that’s really nice. I do like that. It promotes tasks. Like I said, I enjoy that too.” [Life coach]

### Superordinate Theme: Met and Unmet Expectations

#### Workflow Ease, Effectiveness, and Efficiency

One of the initial expectations was that the new care management tool would make the completion of the processes and associated tasks more efficient and effective. Employees’ expectations were met by the tool’s implementation, as it brings all data into an electronic form and under one platform (Textbox 14). Previously, information was collected and stored in various places and different formats:

**Aspiranet staff reflect on their expectations moving to an all-in-one platform.**

- “One of the expectations (we had and) was not an immediate expectation. Something that would happen over time is that there would be kind of efficiencies associated with the work that a life coach did and improvement in their process... Being able to, you know, access data and enter data in the field to have data all in one place, as opposed to in a lot of different (place), you know, systems or paper files or whatever. And I think to that extent, Watson has met those expectations.” [Leader]

#### Augmented Intelligence Using Artificial Intelligence

Artificial intelligence technologies was an area where employees had higher expectations that they felt remained unfulfilled (Textbox 15). They believed that since the technology is still evolving, their expectations would be met in the near future:

**Aspiranet staff reflect on their expectation of an artificial intelligence technology.**

- “You know, I was expecting a robot to be honest almost. Maybe, I was not being realistic that (it) was going to take over (and) is going to help?? (us) through every single step...I had very high expectations of it and maybe unfair expectations of it, you know so I would say that while it has not fully met my entire expectation of it. You know, it’s making steps towards that very slowly...” [Leader]

### Superordinate Theme: Limitations and Recommendations

#### Intuitive Client Dashboard

Having a client dashboard for users and to generate reports enabling them to see their client snapshots is another feature currently absent that would improve the workflow (Textbox 16):

**Aspiranet staff reflect on what potential features like dashboards that would be helpful in the future.**

- “And so, like I said, it, that part has been hard to like (i.e.), to run a quick report...also our supervisors can’t (run reports), there is not like a quick dashboard. They would have to understand tables in order to go into Cognos and run the report to get that information.” [Leader]

#### Enhanced Information Retrieval Functionalities

Integration of a sophisticated search engine to further expedite information retrieval was a commonly reported theme (Textbox 17):

**Aspiranet staff reflect on what potential features like search engines that would be helpful in the future.**

- “If you can search, like, a search window where you can type (such as), if you are looking for some specific doctor’s visit, so type in (doctor’s visit), and it pulls up all the doctor’s visit. Something like that.” [Life coach]

#### Efficient Data Management

Data management, such as automating the data population, the ability to bulk edit, and repeated data entry tasks, is believed to be suboptimal. Additional developments around these features could likely reduce the redundancy of task completion and hence enhance efficiency (Textbox 18):

**Aspiranet staff reflect on efficiencies that could be gained through data automation.**

- “(Getting tasks done) is tedious (having), repetitive forms or repetitive actions and tasks or just having their name and birthday (repeatedly entered) in every form, it just gets to be too much...” [Life coach]

#### Enhanced Communications Functionalities

Some of the recommendations made by the employees were enhancing the communications functionality within the tool; for example, provision of email or chat between supervisors and life coaches for quick communications (Textbox 19):

**Aspiranet staff reflect on how their recommendations for WCM would improve internal communication.**

- “Supervisor needs to review something that a life coach completed, there should be like a way to alert them through the tool. Or I feel like, in this case, it would be an email that needs to be sent saying, ‘hey, you have an alert on this client’.” [Life coach]

### Primary Data: Quantitative Analysis

**Figure 2:**
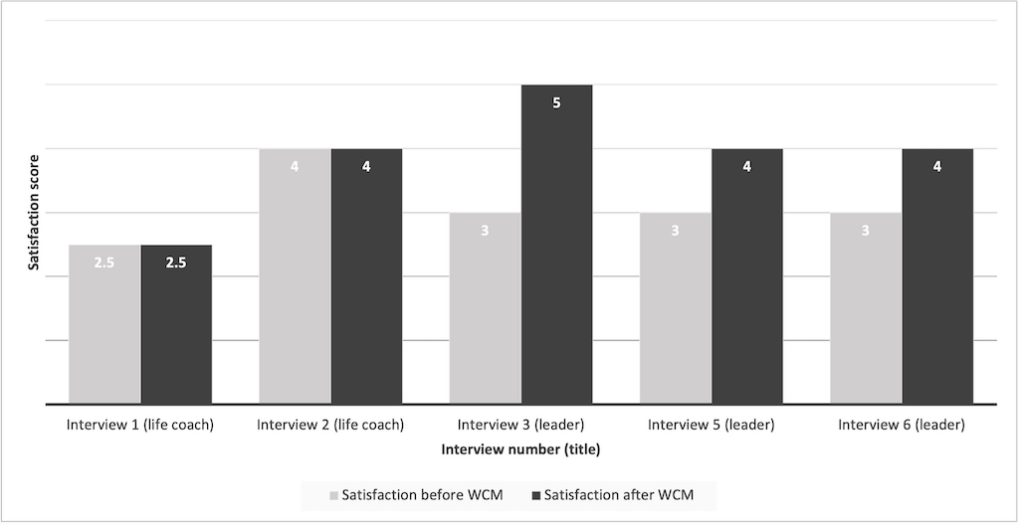
Pre– and post–Watson Care Manager implementation satisfaction scores measured on a 5-point Likert scale. The number denotes self-scored satisfaction.

When comparing pre- and post-WCM implementation satisfaction scores, as measured using a 5-point Likert scale, the scores remained constant between the 2 life coaches (median 3.25, IQR 2.5-4). Among leaders, the results suggest a higher satisfaction score for post-WCM implementation (median 4, IQR 4-5) when compared with pre-WCM implementation (median 3, IQR 3-3; Figure 2). Data from interview 4 were not included because there was no before and after comparison.

### Secondary Data: Quantitative Analysis

#### Youth Demographics, Length of Service Days, School Enrollment, and Employment

Across the 2 study phases, the Aspiranet TAY program served 1641 transitional youth. The median age (IQR) was 18 (18-19) years, and 55.32% (614/1641) of the patients were female. The reported race or ethnicity data across this population included Hispanic or Latino (621/1641, 37.84%), Black (470/1641, 28.64%), White (397/1641, 24.19%), and other (153/1641, 9.32%; reported in Table 2).

**Table 2:** Youth demographics and social determinants of health characteristics pre– and post–Watson Care Manager (WCM) implementation.

Youth characteristic			Pre-WCM (n=531)		Post-WCM (n=1110)
Age at start (years), median (IQR)			18 (18-19)		18 (18-19)
Gender (female), n (%)			289 (54.4)		614 (55.32)
**Race and ethnicity, n (%)**					
	Hispanic or Latino	202 (38)		419 (37.75)	
	Black	152 (28.6)		318 (28.65)	
	White	126 (23.7)		271 (24.41)	
	Other	51 (9.6)		102 (9.19)	
Length of service (days), median (IQR)			234 (130-479)		249 (123-458)
**Current school status, n (%)**					
	Enrolled full time	359 (67.6)		833 (75.05)	
	Enrolled part time	61 (11.5)		91 (8.2)	
	Not enrolled	111 (20.9)		186 (16.76)	
Employed, n (%)			137 (25.8)		152 (13.69)
**Housing status, n (%)**					
	Vacancy (existing)	130 (24.5)		885 (79.73)	

The overall median length of service (IQR; ie, time spent under care management) was 247 (125-468). School enrollment varied, with most youth enrolled full-time (1192/1641, 72.64%), some enrolled part time (152/1641, 9.26%), or some not enrolled at all (297/1641, 18.1%). Youth employment and available housing vacancy were 17.61% (289/1641) and 61.85% (1015/1641), respectively.

Table 2 summarizes the characteristics of the pre- and post-WCM implementation. The data showed that youth demographics and median length of service days remained consistent for both phases. The school status showed some increase in full-time enrollment, a decrease in part-time enrollment, and a reduction in the number of students not enrolled. The percentage of youths employed from the pre-WCM to post-WCM phase declined, likely impacted by the COVID-19 pandemic. With respect to housing, the number of available vacancies increased substantially from before to after the WCM.

#### Incidence of Undesired Events Among Youths

**Figure 3:**
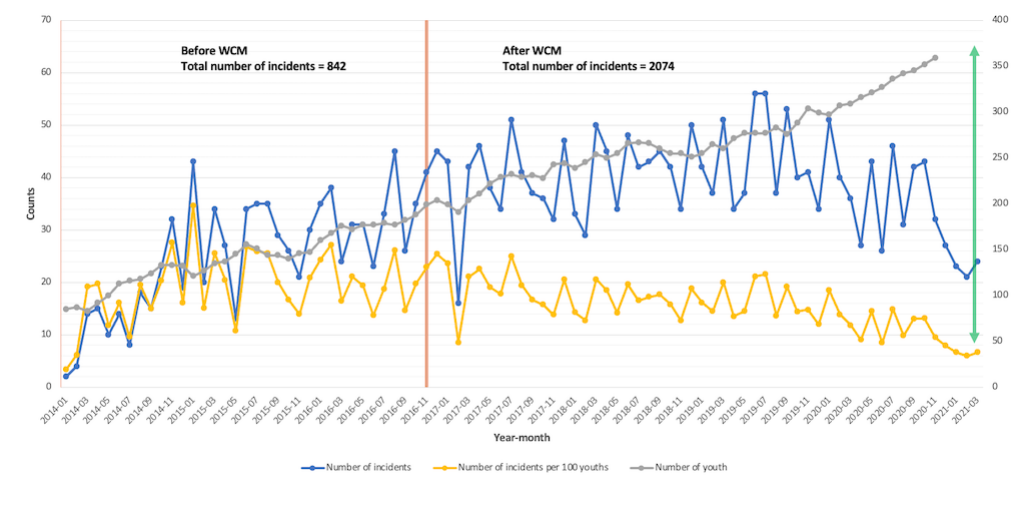
Number of incidents, number of youths receiving services, and number of incidents per 100 youths. The red line delineates the before and after phases, whereas the green arrow indicates the widening gap between the number of youths (increasing) and the number of incidents (decreasing). WCM: Watson Care Manager.

For the 2 phases under study, the number of incidents (undesired events) reported was 842 and 2074 for pre- and post-WCM implementation, respectively. Figure 3 shows the monthly trend for the number of incidents (blue line), number of youths receiving services (gray line), and number of incidents per 100 youths (yellow line). The number of youths receiving services increased over time (gray), while the number of incidents (blue) seemed to increase slightly over time, even though variability was high. The results also suggest that, although there is variability, the number of incidents per 100 youths (yellow) shows a slight decrease over time.

## Discussion

### Principal Findings

Providing extended foster care to help prepare disadvantaged young adults for transition into adulthood is a complex process. TAYs are often confronted with multiple challenges including social, emotional, physical, and mental instabilities (eg, lacking basic life skills, housing insecurities, criminal involvement, and depression or anxiety) simultaneously. Existing processes around managing those processes are often fragmented and resource intensive, which can interfere with effective and efficient care delivery and desired youth outcomes. It is imperative for organizations to take actions to overcome such roadblocks (eg, managing large amounts of typically paper-based data stored in various formats and at disjoint locations or building bridges to integrate existing silos in operational activities between disparate organizations involved in rendering services). The implementation of WCM at Aspiranet to assist in managing the care of youth transitioning from foster care to adulthood has enriched workflow processes and is positively impacting youth outcomes by the transformation from a traditional paper-based system to an integrated and interdisciplinary technology-based system. Using WCM, Aspiranet’s leaders and life coaches can develop integrated care plans for youth under their management. WCM helps in successfully triaging the vulnerable youth population to the optimum services they need in a more effective and efficient manner compared with the former highly manual, paper-based processes. In terms of youth outcomes, the results showed an increase in full-time school enrollment and a reduction in part-time school enrollment. A greater availability of vacant residential options serves as a proxy for favorable youth behaviors. Landlords’ satisfaction is higher and their need has decreased in refuting TAY for housing privileges because of their hostile behaviors. The median number of days spent in the foster care program remained the same; however, the number of incidents reported per month per 100 youths showed a steady decline, even with an exponentially increasing number of enrolled youths (Figure 3).

Analysis of the interview data revealed that the implementation of the tool facilitated streamlining the workflow and efficient retrieval of youth information. The data are accessible anywhere and at any time, in contrast to previous methodologies that require digging through file cabinets and driving miles to access data. Currently, with WCM, information is more accessible and thus more usable for life coaches. Users reported more meaningful and productive interactions with the youth under their management, and the availability of a visual representation of the youths’ progress and accomplishments enhances monitoring. Visualization helps develop hope and motivation among otherwise discouraged youth. Finally, quick access and sharing of information allowed users to respond to emergency situations faster, lowering the risk of undesired outcomes.

As observed in previous studies, we found that when new technology is introduced, there is often a disconnect between leaders and staff as they see things differently [32]. The same applies to the perception of WCM; that is, more favorable responses from leaders than from life coaches and direct users (with more frequent daily use) were identified in a recent study [19]. However, owing to the small sample size, we were unable to make any definite conclusions. Some plausible reasons may be that users showed frustration with repetitive data entry into the tool; the process was not fully electronic and the need for continued paperwork comprised approximately 20% to 30% of the total work; or usability-related challenges (eg, too many tabs that they are not familiar with, lacking functions such as limited formatting options, or missing bulk editing), lack of a dashboard, and a suboptimal reporting system. These learnings can be leveraged and used as opportunities for system improvement.

In terms of youth outcomes, full-time school enrollment increased, whereas part-time attendance declined, indicating that youth were more focused and able to successfully compete their goals. Employment decreased, possibly because of the COVID-19 pandemic, and the state of California revised school attendance laws during the pandemic. No change was observed in the length of service (ie, time spent under care management). The data showed a significant increase in available vacant housing, which could be attributed to WCM. The explanation is that WCM provided a better picture and understanding of the maintenance needs of the apartment complex or residential units, which resulted in a more proactive response to apartment landlord needs and sustained a strong relationship. According to one of the leaders, retaining the existing landlord or apartment relationship is a key factor that allows the program to continue to grow. Without available apartments for placement, youths in the TAY program would not exist, and the foster care program would not be as successful as it is today.

As the number of youths increased, the total number of undesired events such as fights, break-ins, falling grades, and police custody increased, but the monthly number of incidences showed a steady decline. This is promising and indicative of potential improvements in behaviors or actions.

With respect to the interpretation of the abovementioned data, we need to keep in mind that these outcomes could be influenced by a number of other variables. Further analysis of quantitative data is needed to make any claims that the benefits surface from the use of WCM per se. The earnings from this study could be used as a springboard to further enhance future artificial intelligence–based technologies in social care management. This could be achieved by improving and fortifying the tool’s current strengths, identifying gaps from a larger group of diverse stakeholders, and addressing them to ensure that they align with their needs and improve youth outcomes.

### Limitations

The study limitations include data challenges, specifically missing data and incomplete or even absent preimplementation use and outcome comparison data. This further illustrates WCM capabilities for storing and saving data in a more readily accessible and secure manner in a single place that is not previously available. Before WCM implementation, the data were recorded using pencil- and paper-based notes, spreadsheets, and databases with fragmented and missing information. A second limitation was a small interview sample size, especially with life coaches. A third limitation was the inability to interview the youth (vulnerable population) to gain an understanding of their perspectives.

Most importantly, the improvement seen in workflow processes or youth outcomes cannot be fully attributed to the implementation of WCM because there are likely multiple confounders, both known (eg, life coach to client ratio, workload, changes in process and service delivery road maps, and availability of resources including funding) or unknown, which require further investigation.

### Conclusions

Foster care and social service agencies providing support to TAY are challenged by synthesizing large volumes of siloed data (such as health, social, behavioral, and judicial data) to support high-impact decisions. A care coordination approach that is supported by technology has many potential benefits. These include serving as a common, shared platform to consolidate and manage electronic data and to create and track specific goals and corresponding actions. These complex processes are now executed more effectively, efficiency gains are realized in real time, with greater perceived acceptability to end users, and ultimately positive outcomes for these youths to become independent and productive members of society.

## Appendix

Multimedia Appendix 1 Interview transcripts used for the study.

## Abbreviations

CLS: Casey Life Skills
TAY: transitional-aged youth
WCM: Watson Care Manager
