# Supplementary material for: Understanding a Care Management System’s Role in Influencing a Transitional-Aged Youth Program’s Practice: Mixed Methods Study
Source: JMIR Hum Factors. 2022 Dec 16;9(4):e39646. doi: 10.2196/39646 (PMC9804088; doi:10.2196/39646)
Supplement: Multimedia Appendix 1 [file humanfactors_v9i4e39646_app1.docx]

**Appendix A**

**Interview Guide**

**Title:** **Evaluation of an Integrated IBM Watson Care Manager Solution in Providing Services to Transitional Aged Youth**

Thank you for agreeing to be interviewed today. I am part of a research team that is assessing the impact of WCM at Aspiranet on providing services to Transitional Aged Youth (TAY) though a Pre- and Post-implementation analysis focusing on ‘processes’ and ‘outcomes’.

The interview will last between 30 minutes and one hour. Before we get started, I’d like to ask you to sign a consent form stating that you are participating voluntarily. It also states that this interview will be completely confidential, and your name will not be used in any kind of reporting or publishing. We will combine the results from all of our interviews to provide a clearer understanding of perspectives and experiences of care coordination using WCM. We aim to evaluate its impact and explore potential recommendations to strengthen client-centered care management approaches for transitional youth.

I would like to tape-record the interview, if that is alright with you. It will not be linked to your name in any way; but it will be transcribed, with any identifying information removed, so that the research team can better analyze the data. Is that okay?

Do you have any questions? Ok, let’s get started.

| IBM Interviewer |  |
| --- | --- |
| Participant ID |  |
| Date |  |
| Start time |  |
| End Time |  |

**Section A**

**Demographic/Professional information (~ allocated time=2 mins)**

1. What is your age?
2. What is your gender? (if you are OK with sharing)
3. What is your educational background? What year did you graduate from your most recent professional program?
4. What is your role at Aspiranet? Probe:
   - Department name
   - Title
   - Duration of experience in this role, at this organization and overall experience in this field
5. Do have any direct interactions with WCM?, if yes then ask:
6. How would you rate your level of comfort with technology in general?
   - Novice (beginner): You have exposure to the skill and understand basic concepts, but you lack experience.
   - Intermediate: You have experience with and can carry out the skill, but you don't understand advanced concepts. For this level skill, you normally wouldn’t need a qualifier.
   - Expert: You have solid experience and training with the skill and understand advanced concepts.

**Section-B**

**General Information**

***Department Description***

1. What is the mission and vision of the Aspiranet and department you oversee/lead?
2. What programs and/or services does your department provides for Aspiranet?
3. What would you say are the major challenges facing your department at this time? Probe:
   - Financial, Infrastructural, Staff Development, Lack Of Integration/Coordination With Other Departments/Agencies, etc.

***Job Role Overview***

1. Can you briefly describe a typical day in your job and your responsibilities in that context? Probe:
   - Kind supervision and number of people being supervised
2. Do you get job performance feedback and evaluation? Probe:
   - How frequent?
   - What is your performance evaluation based on?

***Client’s Burden Of Care***

1. Can you tell me about transitional youth population characteristics that are currently under Aspiranet care?
2. Can you tell the most pressing challenges your clients face?
   - Probe: Financial, health, social, educational, housing, criminal justice.
3. How does your department help clients address those challenges?

**Section-C**

**Perspectives Around Task Processes And Their Results**

1. At a higher level, can you please describe and compare:
   - The workflow associated with traditional system (Pre-implementation)?

And

- - How workflow it has been impacted by using Watson Care Manager (Post-implementation) ?

Probes:

- What systems/processes do staff/clients need to navigate in order to access services your department provides? (Pre and Post)
- What barriers exist that can make it difficult for your staff/clients to manage/access and/or maintain access to such services? (Pre and Post) e.g., paper forms, many steps, time delays, in person visits to the incidents
- What enablers exist that can facilitate your staff/clients’ manage /access and/or maintain to these services? (Pre and Post)e.g., forms no longer needed, quality improved in touchpoints and notes, more time to focus on youth (frequency of contacts, length of visit, completion of documentation), information no longer needed to memorize (ability to recall and human error, better collaboration improved with WCM, safety and risk assessment streamlined, remote work, before when there was an incident, care manager would have to drive to location, now they can log in remotely, less overtime for staff, remote audit, streamlined intake process, Organized the data, better patient engagement (patient portals).

1. Can you share your insight around impact of WCM implementation on cost, time, and resource savings? Probe:
   - Through “time-to-task” and “reduction in specific tasks” e.g., time savings documenting risk assessment, time savings documenting care plan, time savings from application to receiving social services benefits, time from risk assessment to implementation of action, reduction # of communications and hand offs between team members via WCM, # users, users across different platforms, reduction in time of monthly case documentation
2. Can you please describe care management workflow at more specific, department or service level (focus areas; Casey life skill, Education, Employment) e.g., relevant actions, tool usage in context of use, and goals: Probe:
   - Share examples: Highlighting internal case collaboration that goes from Life Coach all the way to a COO; what the portal may bring; specific case (individual client(s) or workflow) that could be highlighted?; capture risk management - practice and outcomes before and after; common steps used in accomplishing specific goals - for example, all the steps involved in getting a driver’s license .

**Section-D**

**Perspectives Around Client Outcomes**

1. Can you please describe and compare the impact of WCM integration on youth outcomes? We will be using the Foster Care Work Group’s description ^1^ to inquire about well-being for youth transitioning from foster care across three broad areas.
   - - Social, emotional, and physical well-being
     - Safety and permanency
     - Economic success

**Section-E**

**Perceived Usability and Satisfaction**

1. Thinking back, how satisfied were you with the previous way of doing things i.e., before WCM? (On a scale of 1-5 with 1 being least and 5 being most satisfied)
2. How satisfied are you now after integration of WCM (if you have been here for more than 2 years) into the care management process? (On a scale of 1-5 with 1 being least and 5 being most satisfied)
3. Looking back to the time before you started using WCM, what expectations did you have about it?
   - In terms of efficiency and productivity clinical workflow, client care, client outcomes, client satisfaction, staff/team interaction
4. Has WCM met your expectations? Why or why not?
5. What are some commonly used features of WCM?
6. What are some common strength and weakness of WCM tool itself (in terms of design and functionality)?
7. Do you have any suggestions on how to further enhance the process/tool?
8. Is there anything else that you’d like to tell me about WCM that I haven’t asked?

**That’s all the questions I have. Do you have any questions for me? Thank you for taking the time to talk with me today – we really appreciate hearing your perspectives**
